# Incorporators, Early Investors, and Learners: a longitudinal study of organizational adaptation during EBP implementation and sustainment

**DOI:** 10.1186/s13012-020-01031-w

**Published:** 2020-09-10

**Authors:** Rebecca Lengnick-Hall, Cathleen E. Willging, Michael S. Hurlburt, Gregory A. Aarons

**Affiliations:** 1 The Brown School, Washington University, St. Louis, MO USA; 2 Behavioral Health Research Center of the Southwest, Pacific Institute for Research and Evaluation, Albuquerque, NM USA; 3 Suzanne Dworak-Peck School of Social Work, University of Southern California, Los Angeles, CA USA; 4 Department of Psychiatry, University of California, San Diego, La Jolla, CA USA; 5 UC San Diego Dissemination and Implementation Science Center, La Jolla, CA USA

**Keywords:** Sustainment, Adaptation, Community-based organizations

## Abstract

**Background:**

The majority of literature on evidence-based practice (EBP) adaptation focuses on changes to clinical practices without explicitly addressing how organizations must adapt to accommodate a new EBP. This study explores the process of organizational—rather than EBP—adaptation during implementation and sustainment. To the authors’ knowledge, there are no previous implementation studies that focus on organizational adaptation in this way.

**Methods:**

This analysis utilizes a case study approach to examine longitudinal qualitative data from 17 community-based organizations (CBOs) in one state and seven county-based child welfare systems. The CBOs had sustained a child-neglect intervention EBP (SafeCare®) for 2 to 10 years. The unit of analysis was the organization, and each CBO represented a case. Organizational-level profiles were created to describe the organizational adaptation process.

**Results:**

Three organizational-level adaptation profiles were identified as follows: incorporators (*n* = 7), early investors (*n* = 6), and learners (*n* = 4). Incorporators adapted by integrating SafeCare into existing operations to meet contractual or EBP fidelity requirements. Early Investors made substantial organizational adaptations during the early implementation period, then operated relatively consistently as the EBP became embedded in the organization and service system. Learners were characterized by steady and continuous attention to new ways that the organization could adapt to support the EBP.

**Conclusion:**

The profiles demonstrated that there can be multiple effective paths to EBP sustainment. Organizational adaptation was calibrated to fit a CBO’s operations (e.g., size of the program) and immediate environmental constraints (e.g., funding levels). Additionally, organizations fulfilled different functional roles in the network of entities involved in EBP implementation. Knowing organizational roles and adaptation profiles can guide implementation planning and help to structure contract designs that bridge the outer (system) and inner (organizational) contexts. Adaptation profiles can also inform the intensity of the implementation strategy tailoring process and the way that strategies are marketed to organizations.

Contributions to the literature

- This study describes a rigorous methodological approach for examining organizational adaptation as a long-term process.
- Findings illustrate three organizational adaptation profiles that are useful for identifying organizational factors related to EBP sustainment.
- This study treats organizations as dynamic and responsive to contextual and implementation constraints as they are integrating or accommodating a new EBP, which expands our understanding of organizational adaptation during EBP implementation.

## Background

### Adaptation as changes to the evidence-based practice

Dominant definitions emphasize adaptation as the systematic modification of an evidence-based practice (EBP) to fit provider characteristics, organizational contexts, and service settings [1–3]. It is widely accepted that such adaptation is an unavoidable and even beneficial aspect of EBP implementation [1, 4]. Baumann and colleagues provide a framework for understanding EBP adaptation that includes three types of adaptation: changes to content, context, and training and evaluation [1]. Context-level changes involve changing the EBP format (group or individual), setting (outpatient or community-based), personnel (mental health professional or peer specialist), and population (tailoring intervention to a new client group, e.g., ethnic minority groups, veterans) [1, 5]. 

Researchers have relied on this conceptualization of EBP adaptation to expand the literature in several important ways, including systematic reviews that explore methods and reasons for, outcomes of, and guidance around intervention adaptation [2, 6, 7]. Scholars have also proposed a framework for reporting EBP adaptations and modifications and a taxonomy for practice adaptations [3, 8]. Additionally, new research suggests that EBP adaptation should be considered as a value equation in which the needs, preferences, and contingencies of stakeholders at system, organization, provider, and patient levels are taken into account [9]. To date, however, researchers often treat organizations as a “structural factor” that can influence how EBPs are adapted [1]. In the present study, we examine organizational adaptation as an ongoing process and explicitly address the internal changes that organizations make (i.e., their adaptations) to accommodate implementation of new EBPs.

### Organizational adaptation and implementation strategies

An implementation strategy is “a systematic intervention process to adopt and integrate evidence-based health innovations into usual care” [10]. The Expert Recommendations for Implementing Change (ERIC) compilation includes a number of strategies that encourage changes or adaptations to structure and/or process in an implementing organization [11]. Examples include creating new clinical teams, organizing clinician implementation meetings, changing physical structure, enhancing or procuring new equipment, developing quality-monitoring tools, and conducting ongoing training [11]. We conceptualize organizational adaptation as an implementation process that includes both planned and unplanned organizational changes and use of implementation strategies.

### Using strategic management to understand organizational adaptation during implementation

Strategic management is a subfield of organizational research that focuses on “the analysis of internal and external environments of [an organization] to maximize the utilization of resources in relation to objectives” [12]. This type of research is motivated by the notion that organizations must adapt to and influence the external environment to survive, and they do so by pursuing a number of different strategies [13, 14]. Strategic management research explores how organizations achieve competitive advantage and manage both predictable and unpredictable environments [13, 14]. Major literature streams describe how organizations develop and implement strategies and assess the relationship between different strategies and organizational performance [13]. Practically speaking, “The major importance of strategic management is that it gives organizations a framework for developing abilities for anticipating and coping with change” [12]. This is directly relevant to understanding how organizations might adapt (to and with a dynamic external environment) during implementation to achieve EBP sustainment.

### Typologies as a tool for advancing organizational implementation research

Typologies focus on “patterns or profiles rather than individual independent variables” [15]. Since the late 1970s, they have served as influential tools for organizational theory-building in the management literature [15–20]. One of the most widely cited and empirically tested typologies is the four organizational types proposed by Miles and Snow: Prospector, Analyzer, Defender, and Reactor [19]. Prospector organizations embrace change and opportunity, actively seek out opportunities, and create a range of new products [19]. Defender organizations prioritize stability and focus on a limited number of products in a small market sector [19]. Analyzer organizations combine the strengths of Prospectors and Defenders in that they balance the exploration of new market opportunities with the maintenance of a core base of traditional products and customers [19]. Reactor organizations are defined by constant instability that causes them to respond ineffectively to environmental change and uncertainty [19].

It is important to note that this is not just a conceptual exercise, as evident in the wide empirical application of and development of measures for Miles and Snow’s profiles [21–24]. In an example that may be more familiar to implementation researchers, researchers used a multi-item measure of the Miles and Snow typology to identify distinct strategy types that hospitals use [25]. In addition to the original Miles and Snow groupings, they identified new typologies that were particular to hospitals (e.g., “Defensive Analyzer” and “Prospective Analyzer”) [25]. We hope that the development of organizational adaptation profiles can prompt similar measurement development and that profiles can be a new way to understand relationships between organizational patterns and implementation outcomes.

### A holistic and configurational view of organizations

Typologies require a configurational view of organizations in which organizations are viewed holistically and a variety of activities and characteristics can occur within each type [15]. When assessing organizational adaptation that occurs during EBP implementation, it may be tempting to focus on a variable such as organizational readiness. Organizational readiness has been defined as “shared resolve to implement a change (change commitment) and shared belief in their collective capability to do so (change efficacy)” [26]. This can be a useful construct in pre- and early implementation phases for assessing the degree to which organizations are prepared to adopt a new innovation.

 A core assumption that underlies organizational typologies is that there may be a set of different activities, approaches, and characteristics that define types. Therefore, measuring the same pre-determined list of independent variables across organizations and across time (e.g., readiness) may not capture the unique dynamic or static structures and/or processes that explain a given profile or type of organization. To our knowledge, this way of categorizing organizational adaptation has not yet been applied to implementation science.

### A contrast to Glisson’s OSC profiles

Perhaps the closest example in existing implementation literature is the organizational culture and climate profiles that derive from Glisson et al.’s organizational social context (OSC) measure [27]. For example, Williams et al. identified four profiles (comprehensive, supportive, indifferent, and constrained) that describe the organizational cultures and climates of schools implementing EBPs for autism [28]. The OSC profiles have been associated with implementation outcomes, clinician behavior, and service quality, and are the basis of the Availability, Responsiveness, and Continuity model of organizational effectiveness [29]. Additionally, the presence of national OSC profile norms allows organizations to compare themselves to other similar organizations [29]. The OSC profiles inform targeted efforts to improve culture and climate. We aim to use profiles to describe the adaptation approaches that organizations take when implementing and sustaining an EBP.

### The EBP implemented in this study

SafeCare® is a well-established, behaviorally prescriptive, and flexible home visitation EBP designed to prevent child neglect and improve outcomes for children at risk of abuse and neglect [30, 31]. SafeCare home visitors work in families’ homes to improve parent skills in parent-infant and child interactions, home safety, and health [32]. Home visitors, coaches, and trainers achieve and maintain certification, and coaches work closely with home visitors to ensure ongoing adherence to the SafeCare model [32]. Current SafeCare adaptation research focuses on changes to the content and delivery of the intervention or cultural adaptations [33–39]. Aarons and colleagues recently published a taxonomy of SafeCare model adaptations [8]. Adaptation types included changes to process, presentation, dosage/intensity, ordering, and content, as well as forestalling, selective integration, exclusion, and supplementation of material [8]. The present study shifts our focus and extends existing EBP implementation research by examining ways that organizations adapt. We hope that drawing attention to organizational adaptation can provoke a more nuanced and layered conversation about adaptation in implementation research.

### Study goal

The goal of this study is to identify and compare different organizational-level profiles of adaptation documented qualitatively during SafeCare implementation and sustainment. The research question is: How do community-based organizations (CBOs) adapt internally over the course of SafeCare implementation? Our longitudinal qualitative dataset is based on interviews and focus groups conducted among administrators and staff at 17 CBOs in one statewide and seven county-based child welfare systems. Rather than focus our analysis on discreet instances of organizational change, we create composite, comprehensive organizational adaptation profiles based on our prospective data.

We use the Exploration, Preparation, Implementation, and Sustainment (EPIS) framework as a conceptual lens. The EPIS framework draws attention to the inner (organizational) and outer (system) contexts, factors that bridge inner and outer contexts, innovation factors, as well as determinants and mechanisms specific to, and across, implementation stages [40, 41]. EPIS framework determinants consider how organizations may adapt in relation to specific factors (e.g., organizational staffing processes, the system-level outer contexts in which organizations operate, e.g. funding/contracting), and how this context may help explain organizational adaptation. Additionally, we used the EPIS phases to attend to the ways that organizational adaptation develops and changes over time. Lastly, the concepts of structural and ideological fit featured in the EPIS framework may explain why an organization might employ a particular adaptation approach. Structural fit describes the degree to which an EBP (or the process of implementing the EBP) aligns with an organization’s existing profile including roles, responsibilities, resources, and capabilities [40]. Ideological fit describes the ways that an EBP aligns with an organization’s mission, and the values, goals, and priorities of the individuals within it [40]. Lack of structural or ideological fit may prompt organizational adaptation to assimilate (with good fit) or accommodate (requiring organizational change).

## Methods

### Study overview

Organizational adaptation profiles were constructed using a cross-case pattern analysis [42]. The empirical unit, or case, was the CBO [42]. We constructed the case summaries using several sources of data: transcript material, SafeCare manuals, and contracting documents from a previous study [43]. Using the case summaries, we assessed each organization’s adaptation process and then compared patterns of organizational adaptation across the cases. These adaptation patterns became our adaptation profiles. Each organization’s profile label was checked and validated using a visual elicitation tool.

### Study context

This secondary analysis draws from qualitative data collected from CBOs for three prospective mixed-methods parent studies. The parent studies were conducted 2008–2013, 2011–2015, and 2012–2017 and built upon a program of SafeCare effectiveness and implementation research that began in 2005. Inclusion criteria were that CBOs were in systems that achieved SafeCare sustainment status and had at least two time points for the qualitative data collection. Sustainment status was determined by Aarons and colleagues’ criteria based on Stirman et al.’s systematic review of sustainment [44, 45]. “Full sustainment” referred to sites that continued to deliver SafeCare while meeting key fidelity requirements [44]. These sustaining sites had certified SafeCare providers, ongoing coaching and fidelity monitoring, and SafeCare team meetings that adhered to model developer standards [44]. The sample for this study included 17 CBOs in one state (State) and seven county-based systems (County A, County B, etc.).

### Data collection

We drew from individual and small group semi-structured interviews and focus groups with personnel involved in SafeCare implementation at each of the 17 CBOs. The personnel include frontline staff, team leaders and supervisors, and agency executives, administrators and/or leaders. We supplemented these data with interviews conducted among statewide and county administrators in the participating service systems. Interview and focus group questions assessed various aspects of SafeCare implementation and sustainment. Previous studies detailed data collection methods and the foci of interview guides [46–49]. For the 2016 data collection cycle, the first author (RL) developed stem questions and prompts specific to organizational adaptation processes (Additional File 1).

Data collectors in the parent studies digitally recorded, professionally transcribed, de-identified, and checked all transcripts for accuracy. For this analysis, 177 transcripts were analyzed, including 119 individual interviews, 20 small group interviews, and 38 focus groups. Table 1 describes the data types and years collected for each of the eight service systems. We used the analysis of 113 SafeCare contracting documents from Lengnick-Hall et al. to contextualize the transcripts and verify information that participants provided [43].

**Table 1:** Description of data sources

System	Organization(s)	Transcript years	No. individual interviews	No. small group interviews	No. focus groups	Contract doc years	No. contract docs
County A	1, 2, 3, 4	2008–2016	49	2	17	2008–2018	17 (1759 pages)
County B	5, 6, 7	2013–2016	9	1	3	2009–2018	13 (242 pages)
County C	8	2013–2016	4	3	2	2011–2018	18 (274 pages)
County D	9, 10	2013–2016	10	0	1	2011–2018	12 (161 pages)
County E	11, 12	2013–2016	6	2	1	2010–2018	15 (419 pages)
County F	13	2012–2016	8	0	3	2009–2018	12 (403 pages)
County G	14	2013–2016	4	2	1	2012–2018	5 (275 pages)
State	15, 16, 17	2013–2016	29	10	10	2005–2016	21 (606 pages)

The number of qualitative sources and data collection time periods depended on when each CBO took part in the parent studies. Given the specific aims of the parent studies, some CBOs have a greater number of transcripts represented in the dataset than others. Several steps were taken to mitigate this. First, of the 17 CBOs in the sample, two had only one round of qualitative data. RL conducted follow-up interviews for these two CBOs in August and September 2018 to get a second time point. RL asked participants to describe implementation challenges, organizational changes that were made, and plans for sustaining SafeCare. Second, peer debriefing and member-checking activities (described below) provided additional opportunities to learn in more depth about each organization and supplemented the transcripts. Third, participants frequently discussed other organizational and service system actors in their interviews and focus groups. Information about each CBO not only featured the perspectives of individuals in that organization, but also those of other participants familiar with the service system and informed by knowledge of interorganizational networks (as described in the EPIS framework) [40, 41].

A second data source was the most recent SafeCare manuals for home visitors, coaches, and trainers, issued by the National SafeCare Training and Research Center (NSTRC). The home visitor manual included an overview of SafeCare, how to engage clients, and detailed steps for implementing each module. The coach and trainer manuals provided an overview of the key responsibilities and processes for each respective role. The coaching manual directly addressed adaptation. In our study sample, minor practice-level adaptations to the SafeCare model may have occurred, and if made, were vetted by NSTRC.

### Ensuring analytic rigor

We employed several strategies to ensure analytic rigor. Peer debriefing meetings among research team members occurred early in the process. Peer debriefing “is a type of group reflexivity that gives the researcher fresh perspectives and guards against bias” [50]. The purpose of the peer debriefing meetings was to identify any gaps or biases in the initial coding and review coding decisions for each case. There were five peer debriefing meetings (November 2017 to June 2018) that included a qualitative data expert from the parent studies and lead investigators, all of whom had extensive experience with the CBOs and SafeCare implementation issues.

Member checking was used at the end of the process as a way to validate the final organizational profile labels for each case. Member checking also helped to guard against researcher bias and allowed RL to verify the adaptation profiles with a broader group of individuals involved in the parent studies [50]. RL led six member-checking meetings (July 2018 to September 2018) with seven researchers who were highly involved in the parent studies (including CW, MH, GAA) and three practitioners. One of the practitioners served as a SafeCare coordinator and assisted with implementation in five of the eight service systems. The other two were from organizations that only participated in the most recent iteration of the parent study. Meetings occurred in-person, via phone call, and by video conference. Case summaries and profile labels were revised after each meeting.

Detailed written notes were taken at every peer debriefing and member-checking meeting. We also created a comprehensive audit trail that included samples of raw data, memos for each transcript, iterative versions of a codebook, case summary reflections, and initial figures that we made as we began to categorize different organizational adaptation patterns [50].

### Constructing the case summaries

We created case summaries for each organization, which involved assembling and summarizing types of adaptation, contexts for adaptation, and when changes were made to characterize each CBO holistically across the implementation stages [50]. First, RL completed line-by-line initial coding of the 177 qualitative transcripts for content that was potentially adaptation related. Initial coding is more inductive, “provisional, comparative, and grounded in the data” [51]. Coded material was sorted by CBO so that each organization’s codes could be reviewed in peer debriefing meetings [50]. In response to peer debriefing, all transcripts were reviewed a second time using focused coding, a more deductive approach [52]. The qualitative data expert and lead parent study investigators highlighted academic partnerships, referral processes, job roles, and client age as particularly important to understanding this study context. These were the sensitizing topics for the focused coding [51]. During focused coding, we began to move across cases and identify and compare organizational adaptation patterns that could help distinguish the profiles [51].

Transcript coding was supplemented and contextualized with a document review. We reviewed the most recent home visitor, coach, and trainer manuals to see how program developers discussed adaptation. SafeCare manuals helped us determine the boundaries of the intervention, and we structured our review around several questions provided in Table 2. The last row of the table includes potential organizational adaptation, which was our focus. We also used the extensive assessment of contracting arrangements completed in Lengnick-Hall et al. [43] to contextualize how contracts supported and/or constrained SafeCare implementation, if organizations were adapting in response to contracting arrangements, and if changes to contracting arrangements represented a type of adaptation. Each case summary described organizational adaptation in terms of what, when, and how organizational-level changes were made.

**Table 2:** Document review of SafeCare manuals

Document review questions	Examples
How do the SafeCare manuals talk about flexibility?	“SafeCare is structured but flexible in its delivery.” (SafeCare Overview) “…it is important to have flexibility to allow the Provider to discuss other related topics. This balance of structure and flexibility will ensure that the Provider knows what to expect out of coaching while also feeling free to discuss other relevant issues.” (Coach manual)
How do the SafeCare manuals talk about the process of adaptation?	“It is critical that any adaptations that are made do not compromise the model structure or lead to a Provider not maintaining model fidelity.” (Coach manual) “Experience and support from your Trainer will help you understand the parameters by which you can facilitate the Provider’s delivery of the program and what adaptations are appropriate and those that deviate from the core of the model and its research base.” (Coach manual)
What examples of adaptation are provided in the SafeCare manuals?	“Providers may need to adapt a session by changing step order, condensing or expanding the number of sessions, and/or adding additional information or practice time to match the family’s circumstances.” (Coach manual) “SafeCare is still appropriate for families in transitional housing and families experiencing homelessness…If necessary, a mock room can be set up to help the parent learn the process of identifying and removing hazards.” (Provider manual)
What are the SafeCare model standards? (used to identify deviation from these standards)	Client age: 5 years old or younger. Parent-Child Interaction Module (PCI) is for parents of children ages 18 months through 5 years old. (Provider manual) Combining with other services: SafeCare can be conducted by itself or with other services. (Provider manual)
What kinds of potential organizational adaptation are discussed in the SafeCare manuals?	“your agency’s implementation may require more sessions or more frequent coaching.” (Coach manual) “your site’s implementation may require more sessions or more frequent Coach support.” (Trainer manual) “agencies may choose to present the parent with a certificate of completion, either for each module and/or for completing the overall program. You may modify these certificates for your agency and families.” (Provider manual)

### Using the case summaries to identify organizational adaptation profiles

We used a cross-case pattern analysis to develop the organizational adaptation profile labels and sort each case into a profile [42]. When looking across the case summaries, the major patterns that we observed pertained to the scope of organizational changes that were made and at what implementation stage these changes were happening. During the cross-case pattern analysis, we realized that the initial categories of minimal, moderate, and extensive adaptation (interview questions in Additional File 1) did not account for the change in organizational adaptation over time that we were observing in some cases. Our initial profile labels were “Minimal adaptation,” “Adaptation decreases over time,” and “Active adaptation over time.” The next iteration of profile labels was “Steady level of minimal or moderate engagement in adaptation,” “Active adaptation at first, then drops off,” and “Proactive adaptation over time.” Our final profile labels, after extensive conversations with the broader research team, were “Incorporators,” “Early Investors,” and “Learners”. Each case was assigned to one of these profile categories for the member checking described next.

### Confirming the organizational adaptation profiles

**Fig. 1:**
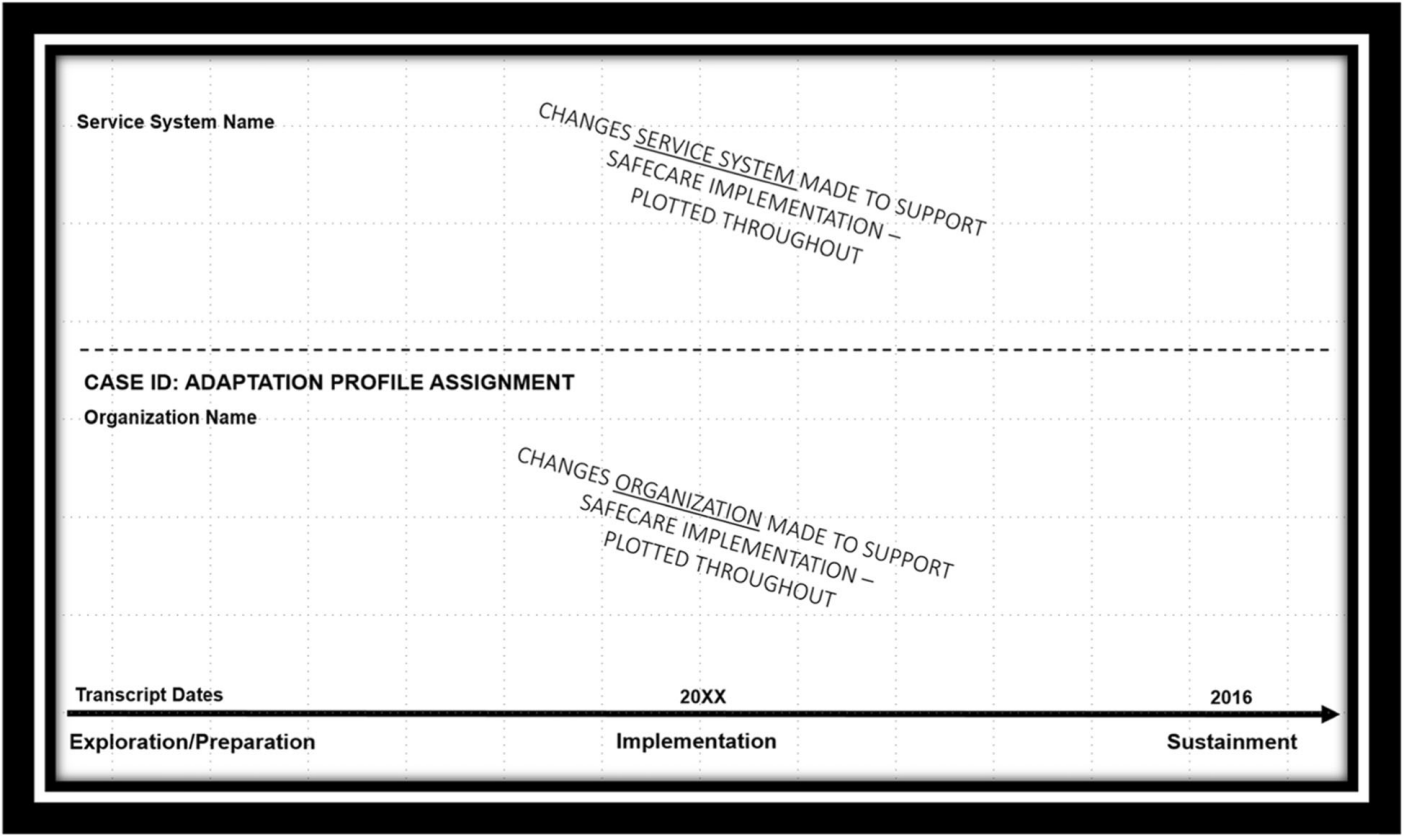
“Adaptation snapshot” visual elicitation tool

We created and used a visual elicitation tool to confirm the adaptation profiles through member checking [50, 53]. During the meetings, specific examples and each organization’s overall approach to adaptation were discussed using a visual elicitation tool “adaptation snapshots” [50, 53]. We created “adaptation snapshots” (Fig. 1) for each CBO. The snapshot had case summary information (e.g., specific changes that they were made and during what implementation stage) as well as an organizational adaptation profile designation. The snapshots were not meant to count every instance of adaptation. Instead, we used the snapshots during member checking to visually describe how the organization adapted, check in about our interpretations of these adaptation processes and examples, review the broader service system context for these adaptations, and verify analytic decisions (including the profile label we applied) [50].

## Results

Table 3 describes participant demographics. Three organizational adaptation profiles were identified, “Incorporators,” “Early Investors,” and “Learners.” Table 4 illustrates the distribution of organizational-level profiles across the eight service systems.

**Table 3:** Participant demographics for qualitative data

	County B (***n*** = 28)	County C (***n*** = 22)	County D (***n*** = 17)	County E (***n*** = 11)	County F (***n*** = 25)	County G (***n*** = 13)	State (***n*** = 92)	Totals^b^ (***n*** = 208)
**Job role**								
Home visitor	10	8	9	5	10	5	46	93
Coach	7	3	0	1	8	1	6	26
Agency leader	7	3	2	3	1	2	12	30
County/state role	3	6	4	1	5	4	10	33
Other^a^	1	2	2	1	1	1	18	26
**Gender**								
Female	24	16	16	8	23	13	74	174
Male	4	5	0	3	2	0	17	31
Missing	0	1	1	0	0	0	1	3

The sample for county A consisted of 133 individuals. However, role, gender, or other identifying information were not collected for this site. As a result, we could not remove duplicates (individuals who participated more than once) or report demographics for this site

^a^Other included supervisor, team leader, and grant writer

^b^Not including county A

**Table 4:** Distribution of adaptation profiles by service system

Service system (***n*** = 8)	Organizations (***n*** = 17)
County A	3 Incorporators, 1 Early Investor
County B	1 Incorporator, 1 Early Investor, 1 Learner
County C	1 Early Investor
County D	1 Incorporator, 1 Early Investor
County E	1 Incorporator, 1 Learner
County F	1 Early Investor
County G	1 Incorporator
State	1 Early Investor, 2 Learners

### Incorporators

There were seven incorporators in five of the service systems. Incorporators were CBOs that “incorporated” SafeCare into existing structures, processes, and capabilities. A key feature of Incorporators was that they primarily integrated the EBP into what they already did as an organization, rather than making substantial changes or creating new ways of operating. This was akin to assimilation. Over time, Incorporators made relatively small-scale organizational adaptations in response to contract and intervention requirements. Small-scale adaptation included adding screening assessments or supplemental material required by the service system to the home visit, adjusting caseloads, adding SafeCare to job descriptions and titles, modifying supervision formats to accommodate SafeCare coaching requirements, and adding the SafeCare program to existing agency forms and documents. As one Incorporator agency leader commented about SafeCare implementation, “…you have to do some things but it’s not overwhelming or hard to do.”

Five of the Incorporators were in a service system that had a dominant lead agency (three within the same service system). One Incorporator agency leader explained the dynamics with the lead agency as, “Over time especially I have really learned how to allow the coaches and to allow [lead agency name] to be the people who they go to for implementation issues.” The major responsibilities associated with initiating, building, and maintaining SafeCare in the system fell on these lead agencies, not the Incorporators.

Six of the Incorporators had contracting arrangements that covered the full costs of implementing SafeCare. An Incorporator supervisor explained, “…I cannot think of anything that needs to be changed for us to better do SafeCare…We have our supplies, we have our transportation, we have our supervision, we have everything...” Another Incorporator agency leader said, “I don’t think there was a need to make extraordinary changes with the resources that were put in place.” Changes related to insufficient funding or lack of resources were not an issue for Incorporators.

Finally, for two of the Incorporators, SafeCare was described as a small part of the agency’s service array. One Incorporator agency leader observed, “It’s a small piece of kind of the tapestry of who we are…our program [SafeCare] has literally one person.” This leader further explained the organization’s adaptation as, “… minimal. Again just the scale of it really wouldn’t drive a lot of heavy lifting changes.” When asked about the percentage of total revenue derived from SafeCare, an agency leader at the other Incorporator organization responded, “A small percentage, very small percentage.”

### Early Investors

Six Early Investors were present in six service systems. Early Investors were CBOs that made substantial adaptations during the initial phases of implementation. Agency leaders at two different Early Investors described this initial effort as “blood, sweat, and tears getting this up and running,” and they “set the stage” for what SafeCare would look like in the entire system. The substantial adaptations that Early Investors made in early implementation were related to the critical system-wide implementation role that these CBOs played. Five of the six Early Investors acted as lead SafeCare agencies in their respective service systems. The sixth Early Investor was initially a lead agency but then became a subcontractor after contracts were renegotiated.

Lead agencies bore significant responsibilities in initiating and maintaining SafeCare in their systems and may have been formally designated as leads in the contracts. Early Investors made strategic adaptations to fulfill a variety of lead agency responsibilities. These responsibilities centered on: (1) coordinating and conducting SafeCare training, coaching, and fidelity monitoring across multiple CBOs; (2) acting as liaisons with service systems, other CBOs, and the NSTRC; (3) managing system-wide SafeCare data; and (4) housing, supporting, and finding ways to pay for at least one SafeCare coordinator. Terms used across the transcripts to describe SafeCare coordinators included “main contact person,” “a resource,” “SafeCare subject matter expert,” “a buffer,” “de facto champion,” and “honorary administrator.”

All of these lead agency responsibilities required substantial adaptation related to developing EBP expertise, creating new processes and procedures, hiring staff or modifying staff roles (especially in the context of training and coaching), and building leadership and data management capacity. These types of changes required greater organizational effort and resources than the way that Incorporators engaged in adaptation.

Additionally, Early Investors changed their approaches to adaptation over time. While Early Investors maintained the organizational changes that they made during early implementation, overall engagement in adaptation tapered off as SafeCare was embedded in the CBO itself and the broader service system. When reflecting on adaptation longitudinally, an agency leader at an Early Investor stated, “I’d say we made extensive changes in the beginning and then minimal changes to sustain it over time.” In this case, the extensive changes had to do with the “entire cultural shift” that had to occur to support SafeCare (described below). An Early Investor team leader at a different agency noted, “Since we’ve been doing this for a few years now, it’s the expected practice…We have a well-oiled machine.” Regarding later implementation stages, an Early Investor supervisor said, “Creativity had been performed already.”

For four Early Investors, SafeCare implementation represented a major cultural shift in the organization. Overcoming ideological misfit and resistance to EBPs generally or SafeCare specifically was part of the early investment that these organizations had to make. Factors that helped to resolve ideological misfit included hiring new staff who were more amenable to the model (one CBO had “massive turnover” as a result of SafeCare implementation), having leaders who made a commitment to “find out what’s good about it and move forward,” creating SafeCare guidelines that could be used when training new staff, and generating buy-in from social workers in the service system who needed to refer clients. Another Early Investor supervisor had to figure out how to integrate providers who had not worked together prior to SafeCare implementation. This supervisor explained, “it was very important for us to get that as one of those elephants in the room, address it, and really have worked strategically to keep our team blended.”

### Learners

Four Learners were present in three service systems. Learners were characterized by steady and continuous attention to new ways that the CBOs could support SafeCare. Learners did not necessarily have more instances of organizational-level changes. Instead, these organizations appeared to have an intangible quality related to a culture and/or leader attitudes that reflected and encouraged curiosity, openness, and a focus on continuous improvement. One Learner agency leader described adaptation in response to SafeCare implementation as, “I want to say we’ve made a lot of changes just to maintain or support the program…we are constantly trying to adapt...” Another Learner leader noted, “I’m committed to knowing or finding out if we’re being effective and wanting to evaluate. I think we have to. We can’t just stumble along in the dark…”

When talking about SafeCare implementation experiences, individuals in Learner organizations used phrases such as: “continually update,” “took the initiative,” “able to quickly adapt or make changes as needed,” “constantly trying to assess and reassess,” “keeps us from being stale,” “constantly tweaking,” “ongoing conversation,” “is there another way we can deliver the service that we haven’t thought about,” and “we’re just ever evolving.” A Learner agency leader explained, “I think there’s just always something to look at through the eyes of how do we sustain and make it part of the overall program versus it’s just something that we do?” Another agency leader noted, “…we’ve fostered or trained ourselves to really value that ongoing learning.” For Learners, SafeCare implementation represented a positive educational experience for the organization’s staff.

Two of the Learners were in stable, well-funded service systems, while two Learners did not have SafeCare contracts that covered full costs of implementing the EBP. One of these Learner agency leaders explained, “…We have to be quite vigilant about keeping up the caseloads and keeping up the presence and the visibility so that we don’t get forgotten about.” The other Learner agency leader with the underfunded SafeCare contract discussed multiple financially related adaptations that the CBO had made, including cross-training staff (beyond the full-time employee covered by the SafeCare contract) and pulling from discretionary funds to cover the costs of SafeCare implementation. Similar to Incorporators, Learners maintained their overall approach to adaptation across the implementation stages outlined in EPIS.

## Discussion

This study used rigorous prospective qualitative methods and analysis to address the unexplored topic of how organizations adapt over the course of EBP implementation. Three organizational-level adaptation profiles emerged: Incorporators (*n* = 7 organizations), Early Investors (*n* = 6 organizations), and Learners (*n* = 4 organizations). The three profiles demonstrate the concept of equifinality or the idea that there can be multiple effective paths to the same outcome [54, 55]. This is the case because all of the organizations in this study successfully moved through implementation to EBP sustainment.

A key feature of the seven Incorporator organizations was that they primarily integrated (or assimilated) SafeCare into existing operations. This is akin to Piaget’s theory of assimilation or accommodation in the learning process but at the organizational rather than individual level [56]. Five of the Incorporators were in service systems with dominant lead agencies. Six of the Incorporators entered into contracting arrangements that covered the full costs of implementing SafeCare. For two of the Incorporators, SafeCare was a small part of the agency’s service array. The Incorporator approach perhaps points to the strategic management concept of beneficial inertia, which occurs when an organization faces minimal (or no) need to change to meet environmental demands [57]. Zajac and colleagues note that internal characteristics, such as possessing “resources that offset external pressures for change” can help determine when inertia is beneficial [57]. Having a lead agency to depend on, adequate resources, and a limited portfolio of SafeCare services may explain why an Incorporator approach most benefitted these CBOs.

Early Investors organizations made substantial adaptations during initial phases of implementation, e.g., leading system-wide language translation of SafeCare materials, training home visitors across the system, and transforming organizational culture to support EBP use generally and SafeCare specifically. Unlike Incorporators and Learners, a key feature of the six Early Investors is that they changed their adaptation approach over time to align with the changing needs of the environment. Overall engagement in adaptation tapered off as SafeCare became embedded in the organization and service system. Five of the six Early Investors acted as lead agencies in their respective service systems, and the sixth was initially a lead agency and became a subcontractor after contract renegotiations. Early Investors played an important system-wide implementation role. This finding points to the importance of examining organizational adaptation within the context of interorganizational networks. In this study, lead agencies were responsible for training, coaching, managing data, and coordinating across other agencies and with system-level leaders. The burden of implementing was particularly high for Early Investors.

Furthermore, Incorporators may have developed because of the presence of Early Investors. Consistent with the EPIS framework construct of interorganizational networks, organizations occupy different spaces in the network of entities involved in EBP implementation [40, 41]. Each organization may fill different functional niches within their system. Knowing a CBO’s implementation role relative to other organizations in the system can help organizational and system leaders collaboratively engage in implementation planning. Planning tasks where the CBO’s role may be important include assessing and allocating resources across the system, identifying service redundancies and gaps, and leveraging interorganizational synergy (e.g., coaching and training across agencies to mitigate turnover and maintain a trained workforce within the system as a whole). This information can also help system leaders craft contracting arrangements that accurately reflect and financially support the different roles that organizations fill.

What characterized the four Learners was steady and continuous attention to new ways that the organization could support SafeCare. The Learner label was inspired by the concept of learning organizations, defined as “an organization skilled at creating, acquiring and transferring knowledge, and at modifying its behavior to reflect new knowledge and insights” [58]. Although developed in the management literature, this concept has been applied to health and mental health contexts [59–61]. Sheaff and Pilgrim assessed whether or not learning organizations can exist in the British National Health System [61]. The authors note, for example, “Team members need to have trust in one another and enjoy the managerial mandate to exploit opportunities as they arise, or experiment with new conditions emerging from the shifting external context that situates the organization” [61]. Learners appeared to have leaders and a culture demonstrating commitment to adapting, improving, and keeping implementation fresh in the organization throughout the implementation lifecycle to reach sustainment. This study was a first step in describing what learning organizations may look like in the context of organizational adaptation to support EBP implementation and sustainment.

Findings can also enhance the implementation strategy literature. Implementation strategy tailoring involves identifying barriers and facilitators, then intentionally selecting strategies that align with these determinants [62]. Knowing an organization’s adaptation profile can help in two ways. First, this information can enable researchers and practitioners to gage the intensity of the tailoring process in advance of implementation. Incorporators, for example, may have a less intense strategy tailoring and selection process than Early Investors during the early implementation phases. Second, researchers can use this information to explain and disseminate implementation strategies in a more targeted way. For example, the package of implementation strategies for an Incorporator may be quite different than the set of strategies that best fits a Learner. Incorporator leaders may be most interested in a small set of strategies that closely align with their current organizational functioning (e.g., updating record systems and revising professional roles). Learner leaders, on the other hand, may want to continually experiment with a variety of strategies that more substantially alter the organization’s core functions (e.g., forming new academic partnerships and accessing new funding streams).

More broadly, we hope that our findings offer a way of thinking about adaptation and organizational research that is new to the implementation science community. Most adaptation research in our field has focused on how to change the intervention [1–9]. Rather than treating organizations as a benign “structural factor that can affect adaptation,” [1] we draw focused attention to the different ways that sustaining organizations approached the process of adaptation. Profiles are a well-known tool in the strategic management literature for understanding how organizations adapt [15–20]. Rather than transporting an existing typology to an implementation study, we wanted to leverage our rich dataset to see what emerged among organizations that were responding to EBP implementation and sustainment.

We recommend the use of organizational profiles (ours and newly identified ones) as a theoretical foundation for empirical testing similar to what has been done with other organizational typologies in the strategic management literature [63]. Recently, Pintello put forth the concept of “organizational phenotypes that promote implementation and sustainment” as a core implementation science principle for future research and one that corresponds with the US National Institute of Mental Health’s (NIMH) current strategic plan [63]. The concept of organizational phenotypes could serve as “diagnostic determinants” for EBP scale-up and sustainment at the organizational level [63]. Profiles, like those identified in the present study, and Glisson’s [27–29] culture/climate profiles, are examples of ways to think about organizational phenotypes for implementation and sustainment research.

### Limitations

This study examined organizational adaptation profiles in the context of SafeCare, one particular EBP. Intervention complexity varies widely, and different organizational approaches to adaptation may emerge with other EBPs. A second limitation is that this study examined organizational adaptation in the context of systems that utilized contractual arrangements to promote and implement SafeCare. Organizations in systems that do not have contractual obligations to implement a particular EBP may engage in other adaptation approaches.

This study used secondary qualitative data to understand adaptation. Social desirability biases and the tenure of participants within a CBO may have influenced the quality of responses. Participants were also recalling and reflecting upon past events. A document review and member checking activities facilitated triangulation of the findings. However, these limitations echo the broader issue that adaptation can be difficult for practitioners to explicitly recognize and report, and accurately assessing adaptation is a persistent methodological issue in implementation science [1].

### Next steps

This study lays the groundwork for multiple avenues of future research. First is the creation of adaptation profiles for other contexts including organizations that do not reach sustainment, are de-implementing a non-evidence based practice, or are implementing a novel EBP. Future research can also explore which implementation activities and strategies are most effective for Incorporators, Early Investors, and Learners. Quantitative methods can be used to further explore characteristics that define the profiles and the relationship between profiles and specific implementation (e.g., fidelity) and client outcomes (e.g., engagement and satisfaction).

Additionally, future research can explore adaptation in the context of the constellation of EBPs that an organization implements. Organizations may use one adaptation approach for one EBP and a different approach for a different practice. For example, some interventions may be a more integral piece of the agency’s total service array (and therefore require a more intense organizational adaptation process). Organizational adaptation profiles may also change in response to environmental threats such as drastic changes in funding, changes in the organization’s mission, or the entrance or exit of key organizational players in the broader systemic network. Or perhaps, an overarching adaptation philosophy may influence organizational adaptation approaches across EBPs.

Future work may also consider the relationship between concurrent organizational adaptation processes, e.g., simultaneous practice, organizational, and system level adaptation that occurs during EBP implementation. Finally, while the importance of cultural and leadership factors for the Learner profile emerged in the qualitative analysis, understanding more precisely how this profile differs from the others requires targeted data collection around published learning organization characteristics.

## Conclusions

Organizational adaptation is an inherent part of EBP implementation, and this study is an essential step in developing theory that allows for the prediction of organizational adaptation behavior during EBP implementation. Successfully implementing a new EBP is one of many tasks that an organization has to attend to, and this study described three different approaches that led to sustainment. Organizations calibrated adaptation approaches to fit their existing scale of operations, structures, and the immediate environmental demands [54, 64]. This study demonstrated that there is no optimal level of adaptation or a singule approach that is necessary for sustainment for all organizations, but rather that adaptation is a dynamic and contextually driven process. This study also  described a rigorous methodological approach for examining organizational adaptation as a longitudinal process.

## Supplementary information

**Additional file 1:.** Adaptation questions included 2016 interview scripts.

## Data Availability

The datasets generated and/or analyzed during the current study are not publicly available. Questions can be directed to the corresponding author (GAA).
