# Supplementary material for: Incorporators, Early Investors, and Learners: a longitudinal study of organizational adaptation during EBP implementation and sustainment
Source: Implement Sci. 2020 Sep 10;15:74. doi: 10.1186/s13012-020-01031-w (PMC7488112; doi:10.1186/s13012-020-01031-w)
Supplement: Supplementary file 1 — Additional file 1:. Adaptation questions included 2016 interview scripts. [file 13012_2020_1031_MOESM1_ESM.docx]

Appendix 1. Adaptation questions included 2016 interview scripts.

1. How do you know if your agency is providing Safe Care as intended by its developers, the National SafeCare Training and Research Center?
2. Are there times the SafeCare model does not work so well?
3. Thinking back since this organization started delivering SafeCare to the present, what internal changes were initiated to facilitate its use? Probes:
   1. Hiring staff
   2. Supervision
   3. Billing
   4. Distribution of workload
   5. Assignment and definition of staff responsibilities
   6. Agency policies and procedures
   7. Referral processes
   8. Staff recognition or rewards
4. Considering some of the changes we just discussed would you say that overall this agency has made minimal, moderate, or extensive organizational adaptations to support implementation and sustainment of SafeCare?
